# Deep Learning Optimizes Data-Driven Representation of Soil Organic Carbon in Earth System Model Over the Conterminous United States

**DOI:** 10.3389/fdata.2020.00017

**Published:** 2020-06-03

**Authors:** Feng Tao, Zhenghu Zhou, Yuanyuan Huang, Qianyu Li, Xingjie Lu, Shuang Ma, Xiaomeng Huang, Yishuang Liang, Gustaf Hugelius, Lifen Jiang, Russell Doughty, Zhehao Ren, Yiqi Luo

**Affiliations:** ^1^Ministry of Education Key Laboratory for Earth System Modeling, Department of Earth System Science, Tsinghua University, Beijing, China; ^2^National Supercomputing Center in Wuxi, Wuxi, China; ^3^Center for Ecological Research, Northeast Forestry University, Harbin, China; ^4^Le Laboratoire des Sciences du Climat et de l'Environnement, IPSL-LSCECEA/CNRS/UVSQ Saclay, Gif-sur-Yvette, France; ^5^School of Atmospheric Sciences, Sun Yat-sen University, Guangzhou, China; ^6^Department of Biological Sciences, Center for Ecosystem Science and Society, Northern Arizona University, Flagstaff, AZ, United States; ^7^Department of Physical Geography and Bolin Centre for Climate Research, Stockholm University, Stockholm, Sweden; ^8^Department of Microbiology and Plant Biology, University of Oklahoma, Norman, OK, United States

**Keywords:** soil organic carbon representation, Earth system model, data assimilation, deep learning, Community Land Model version 5 (CLM5), soil carbon dynamics

## Abstract

Soil organic carbon (SOC) is a key component of the global carbon cycle, yet it is not well-represented in Earth system models to accurately predict global carbon dynamics in response to climate change. This novel study integrated deep learning, data assimilation, 25,444 vertical soil profiles, and the Community Land Model version 5 (CLM5) to optimize the model representation of SOC over the conterminous United States. We firstly constrained parameters in CLM5 using observations of vertical profiles of SOC in both a batch mode (using all individual soil layers in one batch) and at individual sites (site-by-site). The estimated parameter values from the site-by-site data assimilation were then either randomly sampled (random-sampling) to generate continentally homogeneous (constant) parameter values or maximally preserved for their spatially heterogeneous distributions (varying parameter values to match the spatial patterns from the site-by-site data assimilation) so as to optimize spatial representation of SOC in CLM5 through a deep learning technique (neural networking) over the conterminous United States. Comparing modeled spatial distributions of SOC by CLM5 to observations yielded increasing predictive accuracy from default CLM5 settings (*R*^2^ = 0.32) to randomly sampled (0.36), one-batch estimated (0.43), and deep learning optimized (0.62) parameter values. While CLM5 with parameter values derived from random-sampling and one-batch methods substantially corrected the overestimated SOC storage by that with default model parameters, there were still considerable geographical biases. CLM5 with the spatially heterogeneous parameter values optimized from the neural networking method had the least estimation error and less geographical biases across the conterminous United States. Our study indicated that deep learning in combination with data assimilation can significantly improve the representation of SOC by complex land biogeochemical models.

## Introduction

Soil is a vast carbon reservoir in terrestrial ecosystems. It stores more than three times as much organic carbon as terrestrial vegetation does (Ciais et al., 2014). Due to its large reserve, a small change in soil organic carbon (SOC) potentially results in strong regulation of the global carbon cycle and its feedbacks to climate change (Friedlingstein et al., 2006; Luo et al., 2015). It is essential that Earth system models (ESMs) can reliably represent historical and current soil carbon dynamics so that they can realistically predict future changes in the land carbon cycle (Le Quéré et al., 2018).

Presently, soil carbon dynamics simulated by ESMs are highly variable and fit poorly with observations (Luo et al., 2015). Modeled global soil carbon storage differs by up to 6-fold among 11 models in the Coupled Model Intercomparison Project phase 5 (CMIP5) (Todd-Brown et al., 2013). None of the 11 models reproduces the spatial distribution of SOC stocks presented in the Harmonized World Soil Database (HWSD) (Luo et al., 2015). In the Multi-scale Synthesis and Terrestrial Model Intercomparison Project (MsTMIP), an ensemble of 10 terrestrial biosphere models showed a wide range in estimated global SOC storage in 2010, from 425 to 2,111 Pg C (1 Pg = 10^15^ g) (Tian et al., 2015).

It has been suggested that uncertainty in simulating SOC dynamics in ESMs may result from variations in parameterization in addition to model structure and external forcings (Luo et al., 2009, 2016; Brovkin et al., 2013; Lovenduski and Bonan, 2017; Bonan and Doney, 2018). Different parameter values can strongly influence the projection of SOC dynamics among different models or within the same model (Luo et al., 2016). Parameter values in the current generation of ESMs are mostly determined on an *ad hoc* basis and may be derived from the results of field experiments, other models, or informed from scientific or gray literature (Luo et al., 2001). It is largely untested whether or not the *ad hoc* parameterization is representative of the system properties to be simulated. Data assimilation techniques have been used to estimate parameter values from observations (Luo et al., 2003, 2016). Parameter values constrained by data assimilation can improve ESM simulation of SOC compared to default parameter values. For instance, the global representation of SOC distribution in the Community Land Model version 3.5 (CLM3.5) compared to the HWSD database was improved (explained variation increased from 27 to 41%) by constraining model parameters with a Bayesian Markov Chain Monte Carlo (MCMC) data assimilation method (Hararuk et al., 2014).

With the ever-increasing stream of high quality geospatial data and developing data-processing approaches, more comprehensive field-measured data and innovative methods are called in improving the representation of carbon dynamics in ESMs (Reichstein et al., 2019). Although the HWSD has been used to constrain models (Hararuk et al., 2014), it is a data product generated through harmonization of data points from soil survey (FAO/IIASA/ISRIC/ISSCAS/JRC, 2012). The harmonization itself potentially introduces significant errors to the data product. Wu et al. (2019) showed that there is a limited capacity to further improve ecosystem model skill for carbon cycle assessment when using upscaled observational datasets. This finding suggests that methods that can directly utilize point observations rather than upscaled data products are preferable. To avoid any errors introduced in generating maps from point data, it is highly desirable to assimilate the original point data instead of data products to constrain models. Meanwhile, new techniques such as deep learning can improve the performance of ESMs with deluge Earth system data (Reichstein et al., 2019). By constructing computational models with multiple processing layers and allowing the models to learn representations of data from multiple levels of abstraction (LeCun et al., 2015), deep learning techniques have promising applications in Earth system science, such as pattern classification, anomaly detection, regression, and space- or time-dependent state prediction (Reichstein et al., 2019). Exploration is warranted on how to properly employ deep learning techniques in reducing uncertainties of simulated carbon dynamics in ESMs.

This study presents an innovative method, via combined data assimilation and deep learning, that can be used to optimally represent SOC in a complex land biogeochemical model (CLM5) with an extensive dataset of vertical soil profiles across the conterminous United States. We first applied two data assimilation schemes (one-batch and site-by-site) to 25,444 vertical soil profiles with the matrix form of CLM5. In the one-batch data assimilation, we used all the 25,444 profiles as one batch to estimate parameter values of CLM5 (assuming parameters to be spatially constant). In the site-by-site data assimilation, one set of parameter values of CLM5 was estimated from one vertical SOC profile at each site. Three methods were then conducted to represent the spatial and vertical distribution of SOC by CLM5 across the conterminous United States. The one-batch method used the optimized parameter values from one-batch data assimilation for each grid-cell in CLM5 to generate continental SOC distributions. The site-level parameter values optimized in the site-by-site data assimilation were assembled and randomly sampled to generate parameter values for each grid-cell and produce continental SOC distributions (random-sampling method). In the deep learning method, parameter values estimated from the site-by-site data assimilation were maximally preserved for their spatial distributions through a trained neural network so that modeled SOC distributions over the conterminous United States best represent those contained in observations. In particular, we highlighted the potential of hybridizing data assimilation and deep learning in advancing the study of global carbon cycle in the big data era.

## Materials and Methods

### Description of CLM5 Model

The Community Land Model version 5 (CLM5) is the latest version of CLM models (Lawrence et al., 2018). Its soil carbon module is similar to that in CLM4.5 (Koven et al., 2013), except that it has an option to change the number of soil layers from a default of 20. In this study, we used ten soil layers with a vertical transformation among carbon pools from the surface to a maximum depth of 3.8 m as in CLM4.5. The soil carbon component of CLM5 includes carbon transfer among four litter pools (coarse woody debris, metabolic litter, cellulose litter, and lignin litter) and three soil organic carbon pools (fast, slow, and passive SOC) in each layer over 10 layers, totaling 70 pools. The thickness of soil layers increases exponentially from the surface layer (1.75 cm) to deep layers (151 cm), with the total depth of 3.8 m over the 10 layers (Figure S1). Vertical carbon transfer between soil layers only occurs among the adjacent layers and represents both diffusive and advective carbon flux transportation as such caused by bioturbation and cryoturbation. The baseline advective rate of carbon flux is set as 0 m/yr in CLM5 as a default, and this was used for our study as well. A detailed description of belowground biogeochemical processes in CLM4.5 and CLM5 is available in Koven et al. (2013) and Lawrence et al. (2018), respectively.

The original CLM5 was converted to a matrix equation by reorganizing carbon balance equations in the original model to the matrix form (Xia et al., 2013; Luo et al., 2016, 2017). The construction and validation of the matrix equation of model CLM4.5 have been described in details (Huang et al., 2018). The matrix equation has 70 carbon state variables [**X**(t)] and describes carbon transfer among the 70 pools as:

(1) $$\begin{array}{l} \frac{dX\left(t\right)}{dt}=Bu\left(t\right)-A\xi \left(t\right)KX\left(t\right)-V\left(t\right)X\left(t\right) \end{array}$$

where ***B*** is a vector (70 × 1) of partitioning coefficients from C input to each of the pools (unitless), and *u*(t) is C input rate (g C m^−3^ day^−1^). ***A*** represents the allocation coefficients among litter and soil pools (unitless), including the transfer coefficients from four litter pools to three soil carbon pools as well as the transfer coefficients of SOC among soil carbon pools in the same layer. **ξ**(t) represents the effects of environmental variables on decomposition of litter and soil (unitless). It includes scalars of temperature (ξ_T_), soil water potential (ξ_W_), and depth (ξ_D_). ***K*** indicates the decomposition rate of SOC in different litter and soil carbon pools (day^−1^). Furthermore, ***V***(*t*) represents SOC mixing among vertical soil layers through cryoturbation or bioturbation (day^−1^). *t* in parentheses indicates that the corresponding element is time-dependent. The detailed matrix representation of each part of Equation 1 is available in Huang et al. (2018). By assuming a steady state of the carbon cycle in the system ($\frac{d\text{X}\left(t\right)}{dt}=0$), the SOC content of each carbon pool at each layer can be calculated as:

(2) $$\begin{array}{l} X\left(t\right)={\left[A\xi \left(t\right)K+V\left(t\right)\right]}^{-1}Bu\left(t\right) \end{array}$$

### Data Sources

This study used all the SOC profiles in the conterminousUnited States from the World Soil Information Service (WoSIS) dataset. WoSIS (www.isric.org) is a worldwide, quality-assessed, georeferenced soil dataset (Batjes et al., 2016, 2017). For SOC, the depth of vertical profiles varies from the soil surface (at the top of organic or mineral soil material) of the land to a depth of more than 3 meters across sites. The geographical locations of all the soil profiles and the number of layers of each profile are as shown in Figure S2. A total of 26,509 soil profiles with a total of 240,148 layers at different depths in the conterminous United States were available for our study.

In addition, we used the mean annual net primary productivity (NPP), soil temperature, and soil water potential to drive the data assimilation and SOC distribution representation. The NPP data is from the MODIS NPP dataset (DAAC, 2018). We took the mean value from 2000 to 2014 of the record. Mean annual soil temperature and soil water potential were obtained from the output of CLM5 model. After running the model to a steady state by the pre-industrial climate forcing (I1850CRUCLM45BGC), we collected 10-year records of mean annual soil temperature and soil water potential of the conterminous United States. The original CLM5 model was run at the resolution of 1.2° (latitude) × 2.5° (longitude). The final outputs were interpolate into the resolution of 0.5° × 0.5°.

We used two sets of global SOC data, WISE30sec (Batjes, 2016) and SoilGrids250m (Hengl et al., 2017), as references to compare with spatial and vertical distributions of SOC obtained from our study over the conterminous United States. WISE30sec is an updated version of the dataset HWSD (FAO/IIASA/ISRIC/ISSCAS/JRC, 2012), generated by using traditional mapping methods at a resolution of 30 by 30 arc sec. SoilGrids250m is a global gridded soil information dataset generated by using machine learning techniques at 250 m resolution. We took data of SOC content over three depth intervals from these two datasets, 0 to 30, 0 to 100, and 0 to 200 cm. All the original data with high resolution was resampled to a resolution of 0.5 by 0.5 degrees.

### Algorithm of Bayesian Markov Chain Monte Carlo Method

According to Bayes' Theorem, Bayesian probabilistic inversion (Xu et al., 2006; Craiu and Rosenthal, 2014) can be expressed as:

(3) $$\begin{array}{l} p\left(\theta |\text{x}\right)=\frac{f\left(\text{x}|\theta \right)p\left(\theta \right)}{p\left(\text{x}\right)}=\frac{f\left(\text{x}|\theta \right)p\left(\theta \right)}{\int p\left(\theta \right)f\left(\text{x}|\theta \right)d\theta } \end{array}$$

where *p*(**θ**) is the prior probability density function of parameter **θ**, representing the empirical knowledge before the observation. *F*(**x**|**θ**) is the conditional probability density of observations **x** given **θ**. *P*(**x**) is the probability of observations **x**. *p*(**θ**|**x**) is the posterior probability density function of parameters **θ**. For a given set of observation, posterior distribution of parameter **θ** is in proportion with the product of *p*(**θ**) and *f* (**x**|**θ**):

(4) $$\begin{array}{l} p\left(\theta |\text{x}\right)\propto f\left(\text{x}|\theta \right)p\left(\theta \right) \end{array}$$

Given the assumption of normality and dependency of prediction errors, the conditional probability density function of observation **x** on **θ** can be expressed as in Equation 5.1 (Sambridge and Mosegaard, 2002):

(5.1) $$\begin{array}{l} f\left(\text{x}|\theta \right)=Aexp\left(-B\phi \left(m\right)\right) \end{array}$$

(5.2) $$\begin{array}{l} \phi \left(m\right)=\overset{k}{\underset{i=1}{\sum }}\frac{{\left(z_{i}-x_{i}\right)}^{2}}{2\sigma_{i}^{2}} \end{array}$$

where A and B are constants, ϕ is a misfit function (cost function), and **m** represents the model used in this study. The misfit function can be further expressed as Equation 5.2 (Hararuk et al., 2014), where *z*_i_ denotes the modeled results, *x*_i_ represents the observations, σ_i_ is the standard deviation of the observations, and k is the number of observations. Considering the quantitive uncertainty assessment of SOC content is absent in WoSIS dataset, we assumed a standard deviation of 30% of the reported value at each site (Harmon and Challenor, 1997).

We applied an adaptive Metropolis algorithm to generate posterior distributions of parameters (Haario et al., 2001). A parameter chain was generated by a proposal distribution (prior distribution). The newly proposed parameter value would be accepted with an acceptance probability of *p*(θ^*k*−1^*|*θ^*new*^) (Marshall et al., 2004):

(6.1) $$\begin{array}{l} p\left(\theta^{k-1}|\theta^{new}\right)=\text{min}\left\{1,\;\frac{f\left(\text{x}|\theta^{new}\right)p\left(\theta^{new}\right)}{f\left(\text{x}|\theta^{k-1}\right)p\left(\theta^{k-1}\right)}\right\} \end{array}$$

(6.2) $$\begin{array}{l} p\left(\theta^{k-1}|\theta^{new}\right)=\text{min}\left\{1,\text{ exp}\left[-\left(\phi_{new}-\phi_{k-1}\right)\right]\right\} \end{array}$$

where θ^*new*^ is the newly proposed parameter value and θ^*k*−1^ is the (k−1)th accepted parameter value. Equation 6.2 is equivalent to Equation 6.1 after substituting equations in Equation 5 to Equation 6.1. The value of the acceptance probability was then compared with a value *u* which was randomly sampled from a uniform distribution U[0, 1]. Parameter value θ^*new*^ would be accepted if *p*(θ^*k*−1^|θ^*new*^) ≥ *u*; otherwise θ^*k*^ was set to θ^*k*−1^.

We used two proposal distributions (prior distributions) in the adaptive Metropolis algorithm. First, a test run triggered by a uniform distribution was conducted. Parameter values were proposed uniformly within a prior range (Haario et al., 2001; Xu et al., 2006; Hararuk et al., 2014):

(7) $$\begin{array}{l} \theta^{new}=\theta^{k-1}+r\frac{\theta^{\text{max}}-\theta^{\text{min}}}{D} \end{array}$$

where θ^*max*^ and θ^*min*^ are the upper and lower limits of parameter values (Table S1). *R* is a uniformly distributed random variable over [−0.5, 0.5]. *D* is a coefficient controlling the step size of the newly proposed parameter value. When *D* = 5, the maximum step size of the newly proposed parameter values will be 1/10 of the range between the lower and upper limits. Second, a multivariate Gaussian distribution was assumed for parameters in the formal run of the adaptive Metropolis algorithm. The newly proposed parameter values would depend on (k−1)th accepted parameter values θ^*k*−1^ as means of the multivariate Gaussian distribution and covariance C_t_ = C_t_*(*θ_0_*, …*, θ_*t*−1_):

(8) $$C_{t}=\{\begin{array}{cc} C_{0} & t<t_{0}\\ s_{d}cov\left(\theta_{0},\;\ldots ,\;\theta_{t-1}\right)+s_{d}\varepsilon I_{d} & t\geq t_{0} \end{array}$$

where covariance matrix C_0_ was calculated based on the accepted parameter values after the test run, *s*_*d*_ is a coefficient that depends only on the dimension of parameters *d*, which was set to be 2.38^2^/*d* for the theoretically most effective acceptance rate of 0.234 (Gelman et al., 1996; Rosenthal, 2011), ε is a constant with a very small value, and *I*_*d*_ represents the d–dimensional identity matrix. The index *t*_0_ > 0 was selected for the length of an initial period in the formal run.

Theoretically, the adaptive Metropolis algorithm has correct ergodic properties (Haario et al., 2001). Therefore, the parameter value chain created by adaptive Metropolis algorithm can converge to a unique stationary distribution (Spall, 2005; Xu et al., 2006). In our study, we used the Gelman-Rubin (G-R) diagnostic method to test the convergence of three independent runs. If parameter chains have reached convergence, the within-run variation should be roughly equal to the between-run variation (Gelman and Rubin, 1992). The within-run and between-run variation are expressed as:

(9) $$\{\begin{array}{c} B_{i}=\frac{N}{K-1}\sum_{k=1}^{K}{\left({\bar{c}}_{i}^{.,k}-{\bar{c}}_{i}^{.,.}\right)}^{2}\;\\ W_{i}=\frac{1}{K(N-1)}\sum_{k=1}^{K}\sum_{n=1}^{N}{\left(c_{i}^{n,k}-{\bar{c}}_{i}^{.,k}\right)}^{2} \end{array}$$

where *i* denotes parameters investigated in this study, *K* is the number of parallel runs, *N* is the length of each run, $c_{i}^{n,k}$ represents the nth accepted value of parameter *i* in the kth parallel run after the burn-in period, and the length of burn-in period was set to be half of the accepted parameter chain. The G-R statistics is then defined as:

(10) $$\begin{array}{l} GR_{i}=\sqrt{\frac{W_{i}\left(N-1\right)/N+B_{i}/N}{W_{i}}} \end{array}$$

Once the convergence is reached, *Gr*_*i*_ should approximately approach 1.

### Methods of Generating SOC Distributions

**Figure 1:**
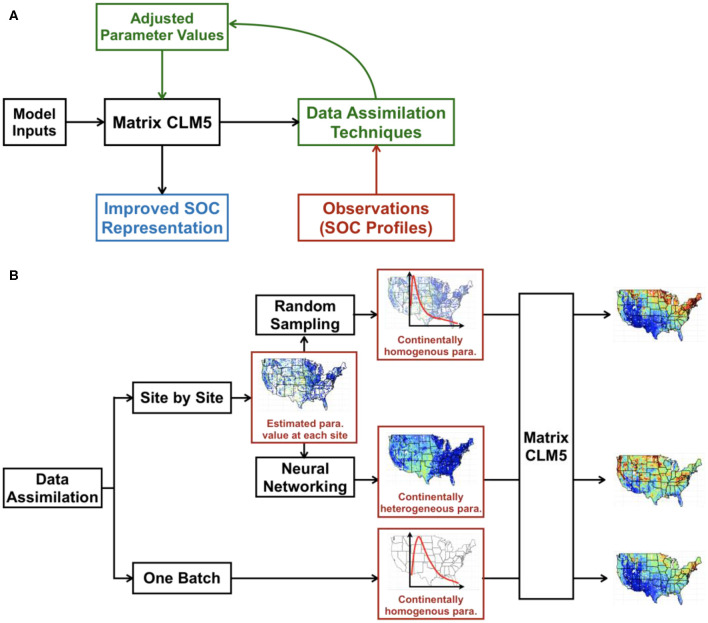
Schema of integrating big data into SOC distribution maps by CLM5. In data assimilation **(A)**, we used NPP, soil temperature, and soil water potential as model inputs to drive the CLM5 model. The model output (i.e., analytical solution of vertical SOC content calculated through Equation 2 under the steady state assumption) will be compared with the observed SOC content in the data assimilation framework, the results of which will further guide the adjustment of parameter values of CLM5 to improve model representation of SOC distribution. The figure was adapted from https://www.hzg.de/institutes_platforms/cosyna/models/data_assimilation/index.php.en. Two data assimilation methods were used and were further developed to generate SOC distribution maps across the conterminous United States **(B)**. Optimized parameter values at individual sites in the site-by-site method were either randomly sampled to generate the continentally homogeneous parameter distribution (i.e., all the sites use the same posterior parameter distribution to generate SOC distribution) or used in training, validating, and testing a neural network to predict parameter values at each grid of the map (continentally heterogenous parameter). The one-batch method can directly generate the continentally homogenous parameter distribution by taking all observational SOC profiles as one batch in data assimilation. The continentally homogeneous distributions by the random-sampling method and one-batch method and gridded maps of parameters by the neural networking method were then applied to CLM5 to generate the SOC distribution of the conterminous United States.

We explored three different methods to optimize parameter values in CLM5 and generate the spatial and vertical distributions of SOC across the conterminous United States. Two data assimilation schemes (one-batch and site-by-site) were first applied. The one-batch method was then applied directly based on the results of one-batch data assimilation. The random-sampling and neural networking methods were conducted based on the results of site-by-site data assimilation. The workflow is as shown in Figure 1.

#### Data Assimilation by One-Batch and Site-by-Site

The one-batch data assimilation used all the observations of SOC content as one batch in the MCMC process (as described in section Algorithm of Bayesian Markov Chain Monte Carlo Method) to constrain parameter values. Three parallel MCMC chains each containing 50,000 iterations as test run and 200,000 iterations as formal run were generated. To effectively capture the vertical distribution pattern of soil content along the depth, we put weights to observations at different depth in calculating the discrepancy between modeled and observed SOC content (i.e., cost function). The weight values assigned to observations at different soil layers decreased exponentially with the depth (i.e., $weight_{i}=e^{-|depth_{i}|}$, where *i* refers to the layers along the soil depth in observations) except for the top layer and the bottom layer, where a weight of 10 was assigned to accelerate calibrating the upper and lower bounds of SOC distribution curve. Meanwhile, to monitor the efficiency of MCMC process, a acceptance rate threshold was characterized. For Markov chains whose acceptance rate was higher than 50% or lower than 15%, the corresponding data assimilation results were rejected (Roberts et al., 1997; Roberts and Rosenthal, 2001). After the MCMC process, we first discarded the first half accepted parameter values of the formal run as burn-in. The G-R statistics for each parameter was then calculated. We randomly selected one Markov chain after eliminating the burn-in period to generate the posterior distribution for each parameter.

The site-by-site data assimilation constrained parameter values of CLM5 with one data set of a vertical SOC profile at each site with the MCMC process. Three parallel chains each containing a test run of 20,000 iterations and a formal run of 30,000 iterations were generated. Weights at different depth in calculating cost function and acceptance control were the same as those in the one-batch method. After the MCMC process, the first half of the accepted parameter values in the formal run were discarded as burn-in. The G-R statistics of each parameter was then calculated for each soil profile. Meanwhile, we randomly selected one chain after eliminating burn-in period to generate the posterior distributions of parameters. For each soil site, we randomly sampled parameter values from the posterior distributions of parameters for 500 times and applied them into CLM5 matrix model to estimate the vertical SOC content distribution. The final estimations were the average of all sampling results.

In the site-by-site data assimilation, to evaluate the effectiveness of data assimilation, the coefficient of determination (*R*^2^) in linear regression of modeled against observed SOC (i.e., *SOC*_*mod*_ vs. *SOC*_*obs*_) was calculated for each soil profile after MCMC. Coefficients of the linear model were fixed, where the slope is 1 and the intercept is 0. In this context, negative *R*^2^ values indicate the effect of estimations on SOC content after data assimilation is even worse than taking the average observed value as predictions. In this study, we took profiles having negative *R*^2^ values as invalid and hereby discarded them from the whole dataset. Moreover, at those sites where only one observational data was available along the depth of the soil, we did not apply the data assimilation to the data point. After those data sets were excluded, 25,444 out of 26,905 soil profiles were used in our study, which accounted for 94.57% of the entire dataset.

#### One-Batch Method

After the one-batch data assimilation, we randomly sampled parameter values from the posterior distributions 1,000 times and applying the sampled parameter values to CLM5 matrix model. We estimated SOC content distributions at different sites by calculating the average of the results. Meanwhile, we provided the uncertainty of simulation by showing the 5 and 95% quantiles of the 1,000 simulations. The same sampled parameter values were further assigned in CLM5 to estimate SOC content distributions at each grid on the map of the conterminous United States at a resolution of 0.5 degrees.

#### Random-Sampling Method

After site-by-site data assimilation, the Maximum Likelihood Estimates (MLE) of parameters' posterior distributions were identified at each site, assuming the posterior probability distribution is generalized extreme distributed (Xu et al., 2006; Hararuk et al., 2014). We assembled MLE values from the 25,444 profiles to build the probability density functions of parameters at the continental scale (i.e., continentally homogeneous distribution), from which we randomly sampled parameter values for 1,000 times to simulate the SOC content distribution in CLM5 matrix model at each soil profile. The average value of sampling outcomes was taken as the final simulation result. Same with the one-batch method, the uncertainty of the simulation results was given by the 5 and 95% quantiles of the 1,000 simulations. We also applied the same sampled parameter values in CLM5 to generate the vertical and spatial SOC content map for the conterminous United States at a resolution of 0.5 degrees.

#### Neural Networking Method

Both the random-sampling and one-batch methods assume that parameter values are spatially homogeneous (constant parameter values across the continent). In reality, parameter values in Equation 1 may be spatially heterogeneous. We hybridized neural networking with the results of site-by-site data assimilation to search for spatially heterogeneous parameter values so that modeled SOC can fit best with observed SOC. We set the mean values of parameters' posterior distributions obtained from the site-by-site data assimilation as target parameter values (output in the neural network). By including 60 environmental covariates including climate variables and land cover types as input, we explored predicting parameter values at different sites by a trained neural network. The detailed list of environmental covariates, as well as the data sources, are as listed in Table S2.

We designed the neural network as containing four hidden layers with backward propagation. The node numbers for each hidden layer were 256, 512, 512, and 256. For each hidden layer, the drop ratio was set as 0.2. The activation function for hidden layers was RELU. We used mean squared error as the loss function and Adadelta as the optimizer. Eighty percentage of the results from the site-by-site data assimilation were used in training and validating the neural network with a validation split ratio of 0.2, and the remaining 20% of the site-by-site data assimilation results were used as the testing set. The number of epochs was set as 400, and the batch size was 64. After the neural network training and validation, parameter values for soil sites in the testing set were predicted based on the trained neural network and corresponding auxiliary environmental covariates. The predicted parameter values were then assigned into CLM5 to estimate the SOC content distributions at corresponding sites. Moreover, to represent SOC content distributions for the whole conterminous United States, we used the trained neural network and auxiliary environmental covariate masks of the continent at a resolution of 0.5 degrees to propose parameter values at each grid of the map. The map of SOC content for the conterminous United States was generated by applying the neural network based parameterization into CLM5 matrix model.

## Results

### Model Representation of SOC Content Across Observation Sites

**Figure 2:**
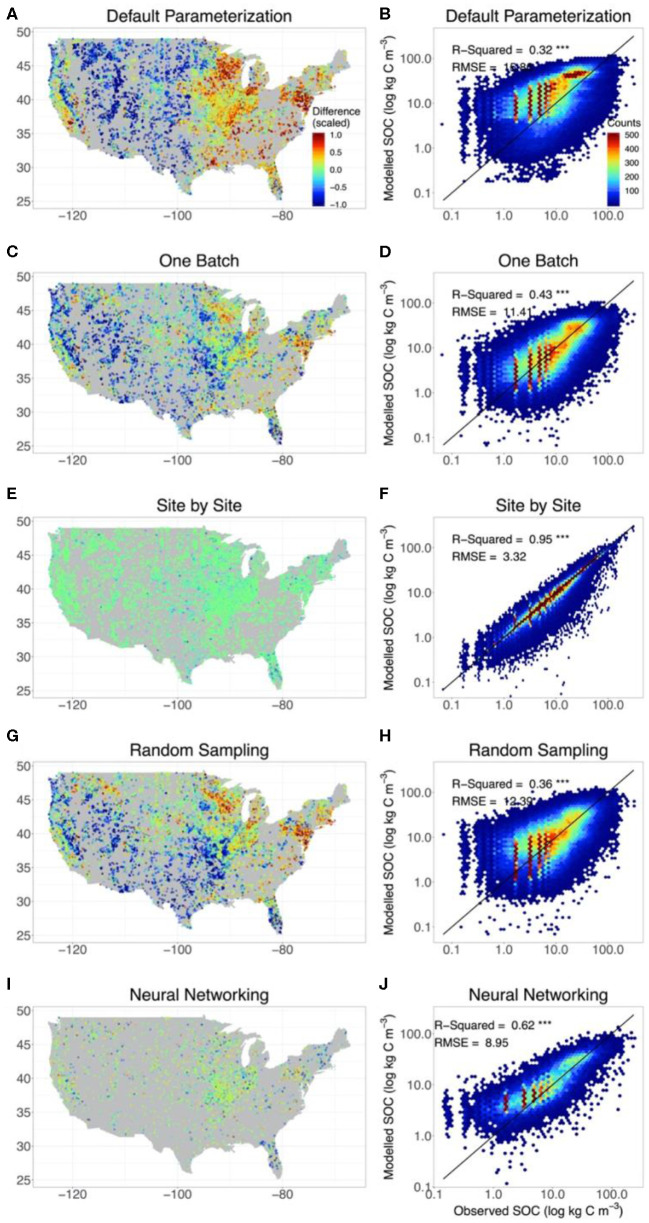
The agreement between observed and modeled SOC content with different methods. For comparison, we also include the results from CLM5 with default parameterization **(A,B)** and from the site-by-site data assimilation **(E,F)**. SOC estimates modeled by CLM5 at different depth of the model setting were extrapolated to the depths of observations to evaluate model performance. The left panels indicate the deviation of the modeled SOC storage from the observation of the whole profile for each site. The right panels show the results of the linear regression between observed and modeled vertical SOC content at different depths in different methods. In calculating the deviation of modeled SOC storage from observations, for better presentation, the positive (overestimation) and negative (underestimation) discrepancy between the observed and modeled SOC content were scaled based on the 95% quantile of the positive discrepancy and 5% quantile of the negative discrepancy, respectively. Meanwhile, only the results of the training set were presented in neural networking method. The SOC content was presented by log axes.

The original CLM5 model with default parameterization presented significant geographical biases on the estimation of SOC content in comparison with observations. Modeled SOC in the grid in which the site of observation was located was compared with observations (Figure 2A). SOC storage was systematically overestimated by the original model on the east and west coasts of the conterminous United States but underestimated in the Midwest. The consistency between observed and modeled SOC content was low, with *R*^2^ = 0.32 and RMSE = 15.9 kg C m^−3^ (Figure 2B and Table 1).

**Table 1:** Performance of CLM5 in representing SOC distribution under different methods.

**Methods**	**Model performance**		
	***R*^**2**^**	**r**	**RMSE (kg C/m^**3**^)**
Default	0.32	0.57	15.86
One Batch	0.43	0.65	11.41
Site by Site	0.95	0.98	3.32
Random Sampling	0.36	0.60	12.39
Neural Networking	0.62	0.79	8.95

*The results from the default parameterization in CLM5 and the site-by-site data assimilation were also included as references. R^2^ is the coefficient of determination in linear regression between the observed and modeled SOC content. r is the Pearson correlation coefficient between the observed and modeled SOC content. RMSE is the root mean square error*.

The one-batch method was designed to generate the distribution of SOC from continentally homogeneous posterior distributions of parameters from assimilating all the observation data once in data assimilation. Using the one-batch method, the mismatch between observed and modeled SOC content in the original CLM5 model was moderately reduced in the north and east parts of the conterminous United States (Figure 2C). However, geographical biases in model representation of SOC were not eliminated. The one-batch method underestimated SOC storage in the Intermontane Plateaus and southern Great Plains (see Figure S3 for physiographical regions of the conterminous United States). Meanwhile, overestimation still existed in the Great Lakes areas and the northeast conterminous United States. Overall, the one-batch method explained 43% variation in the observed SOC content with RMSE = 11.4 kg C m^−3^ (Figure 2D and Table 1).

In the site-by-site data assimilation, the optimized parameters at individual sites can fit modeled SOC storage with observations very well. No obvious geographical biases occurred across the conterminous United States (Figure 2G). 95% variation of the observed SOC content was explained by CLM5 through the site-by-site data assimilation, and the RMSE was reduced to 3.3 kg C m^−3^ (Figure 2H and Table 1).

Similar to the one-batch method, the random-sampling method retrieved continentally homogeneous posterior distributions of parameters by assembling estimated parameter values in site-by-site data assimilation. Overestimation of SOC storage in the northeast and underestimation in the southwest of the conterminous United States still exist in this method (Figure 2E). Across observational sites, the random-sampling method explained 36% variation in the observed SOC content with RMSE = 12.39 kg C m^−3^ (Figure 2F and Table 1).

Through a trained neural network with site-level environmental covariates, the neural networking method obtained the optimized parameter values at each site across the conterminous United States. This method achieved a better representation of SOC distribution than the random-sampling and one-batch methods. No systematic geographical biases in estimating SOC storage were observed across the conterminous United States (Figure 2I). The modeled and observed SOC content were highly correlated with *R*^2^ = 0.62 and RMSE = 9.0 kg C m^–3^ (Figure 2J and Table 1).

### Spatial Distribution of SOC Across the Conterminous United States

**Figure 3:**
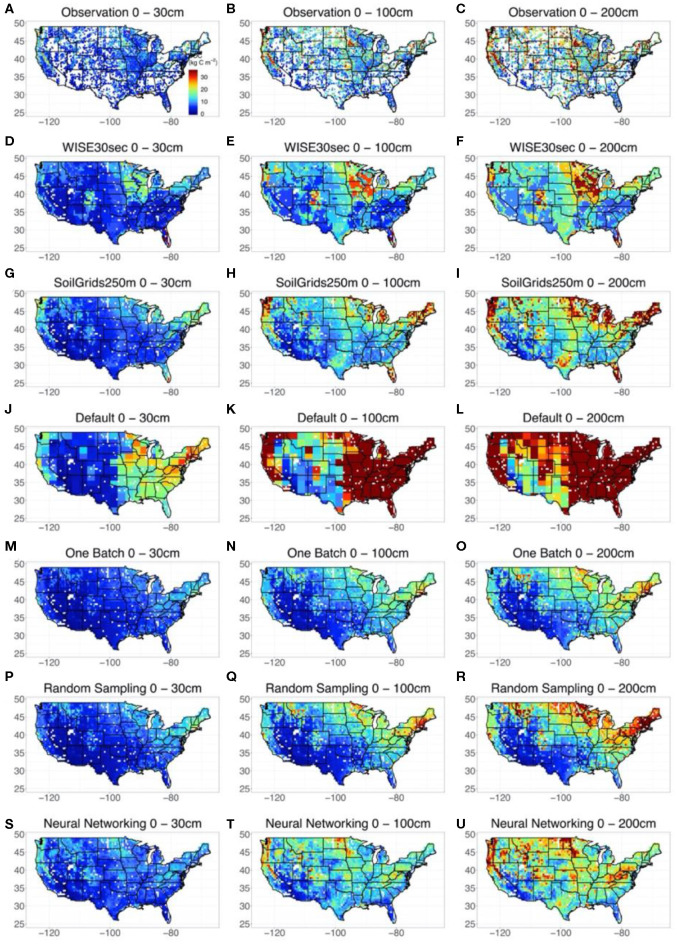
Modeled spatial and vertical SOC distribution across the Conterminous United States by different methods and datasets. Three depth intervals were presented, which were 0–30, 0–100, and 0–200 cm respectively. Because the site-by-site method obtained an almost linear agreement between observed and modeled SOC content, the estimations of SOC content distribution at each site by the site-by-site method was taken as observations for comparison. The results of WISE30sec and SoilGrids250m were also presented as references. The uncertainties of the simulation by the random-sampling method and one-batch method were shown in Figure S4.

We took point observations (Figures 3A–C) and estimations from WISE30sec (Figures 3D–F) and SoilGrids250m (Figures 3G–I) as references for the SOC representation by default CLM5 model and the methods explored in this study. At the continental scale, all the references suggested large volumes of SOC in the northeast and northwest of the conterminous United States The magnitude of SOC content in these regions could be as high as 30 kg C m^−2^ for the 0 to 200 cm depth interval. Meanwhile, a gradient of SOC with decreasing content from the northeast to the southwest was also observed. In sub-regional representation, high SOC content existed in areas across the Great Plains, extending from Texas to the Great Lakes.

In representing the spatial distribution of SOC across the conterminous United States, the default CLM5 model (Figures 3J–L) captured the continental SOC content gradient from the northeast to the southwest but failed to reproduce sub-regional features of SOC distribution in the Great Plains. Meanwhile, SOC content in the east and northwest conterminous United States estimated by the original CLM5 was significantly higher than that indicated by the references. Using data assimilation, both the one-batch method (Figures 3M–O) and random-sampling method (Figures 3P–R) reproduced the continental SOC content gradient from the northeast to the southwest with reasonable values. However, high SOC content in the Great Plains was still not properly represented by these two methods. Significantly, the neural networking method hybridized with data assimilation helped CLM5 mapped the most comprehensive spatial SOC distribution in this study (Figures 3S–U). In addition to capturing the continental SOC distribution pattern, the neural networking method presented relatively accurate sub-regional SOC distribution patterns in the Great Plains, similar to the references.

### Vertical Distribution of SOC Across the Conterminous United States

**Figure 4:**
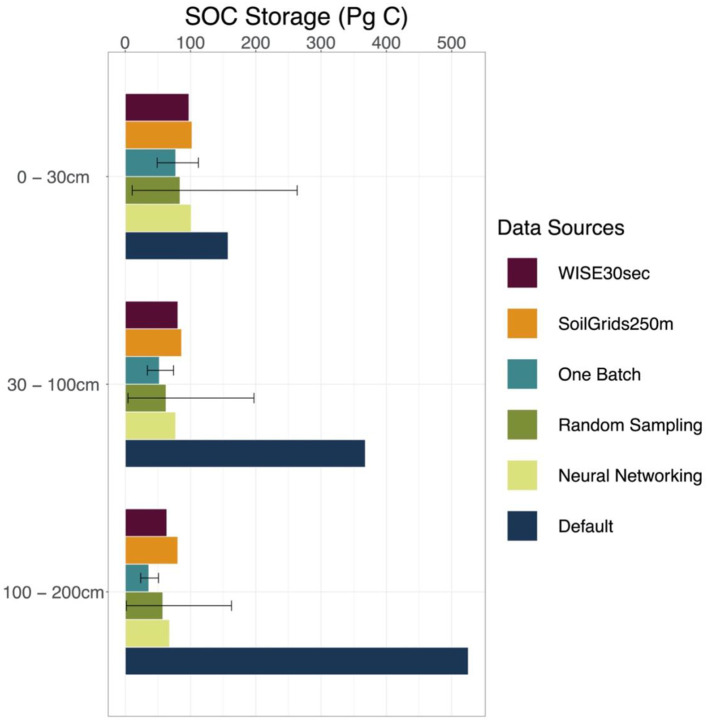
SOC storage estimated by different methods and data sources. Estimations on the SOC storage of the conterminous United States by different methods discussed in this study (one-batch, random-sampling, and neural networking methods) and data sources (original model output with default parameterization, WISE30sec, and SoilGrids250m) were presented by three depth intervals, which are 0–30, 30–100, 100–200 cm, respectively. The error bars for the random-sampling method and the one-batch method present the intervals between the 5 and 95% quantiles in the 1,000 simulations.

We took results from WISE30sec and SoilGrids250m as references in estimating SOC stocks at different depth intervals (Figure 4 and Table S3). Along the soil depth, WISE30sec suggested 98 Pg C at the 0 to 30 cm interval, 81 Pg C at the 30 to 100 cm interval, and 64 Pg C at the 100 to 200 cm interval. SoilGrids250m estimated 102, 86, and 81 Pg C at 0 to 30, 30 to 100, and 100 to 200 cm depth intervals, respectively.

The original CLM5 model with default parameterization substantially overestimated SOC stocks than the references in all three soil depth intervals. CLM5 with default parameterization estimated 158, 368, and 526 Pg C at 0 to 30, 30 to 100, and 100 to 200 cm depth intervals, respectively (Figure 4 and Table S3). Compared with the references, the overestimation on SOC storage made by the original CLM5 model becomes bigger with increasing soil depth.

All the methods used in this study (the one-batch, random-sampling, and neural networking methods) estimated more reasonable SOC storage of the conterminous United States than the original CLM5 model along the soil depth (Figure 4 and Table S3). At the 0 to 30 cm interval, we estimated $77_{49}^{112}$ Pg C using the one-batch method, $84_{11}^{263}$ Pg C using the random-sampling method, and 101 Pg C using the neural networking method. At the 30 to 100 cm interval, the values for SOC storage estimated using the one-batch, random-sampling, and neural networking methods were $52_{34}^{74}$, $63_{4}^{197}$, and 77 Pg C, respectively. At the 100 to 200 cm interval, the estimations were $36_{24}^{51}$ Pg C using the one-batch method, $58_{2}^{163}$ Pg C using the random-sampling method, and 68 Pg C using the neural networking method. As a more robust method, the one-batch method holistically considered all the observations in one optimization algorithm instead of simply assembling individual optimization results, and therefore presented less uncertainty than the random-sampling method in model simulation.

**Figure 5:**
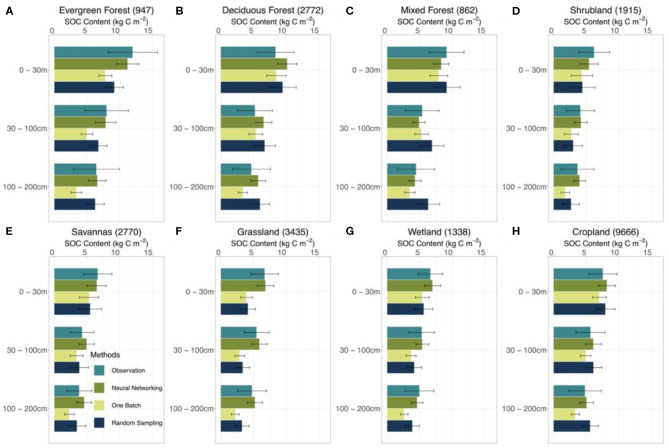
Estimated SOC content in different vegetation types by different methods. Model performance at 0–30, 30–100, 100–200 cm depth intervals by different methods (i.e., neural networking, one-batch, and random-sampling methods) was presented. The observation bars were plotted from the results of the site-by-site method considering its very good linear agreement between observed and modeled SOC content. The number in the parentheses after the names of vegetation types suggests the number of sites at corresponding vegetation type in the dataset we used in this study. The error bars indicate the ±0.5 standard deviation. The distribution of different vegetation types on the conterminous United States is shown in Figure S7.

In different vegetation types, the neural networking method presented more accurate estimations of the vertical SOC distribution than the other methods (Figure 5). Both the one-batch and random-sampling methods underestimated the SOC content in the evergreen forest, shrubland, savanna, grassland, and wetland regions along the soil depth. The neural networking method, in contrast, presented the least biased estimations in comparison with observations at all depth intervals in the aforementioned regions.

### Posterior Probability Density Functions of Parameters After Data Assimilation

The Gelman-Rubin (G-R) statistics for all the parameters were evaluated for convergence of the site-by-site method and one-batch method (Table S1). The G-R values approaching 1.0 indicated the repeatability of the posterior distribution for each parameter in the MCMC process for the site-by-site method at individual sites and the one-batch method (Craiu and Rosenthal, 2014).

In the random-sampling method, the assemblages of the MLE values for most parameters were constrained to their narrow ranges (Figure S5, yellow curves). However, some parameters were poorly constrained after the MCMC process in the site-by-site method as their variances were quite large. Parameters such as *tau4l2l3, tau4l1*, and *fs2s1* (see Table S1 for the functions of parameters in CLM5) tend to have flat posterior distributions, with mean variance being larger than 0.06 across the continent (Table S1). Conversely, parameters such as *tau4s2, tau4s3*, and *efolding* had mean variance not larger than 0.04 in the site-by-site method, which indicated the posterior distributions were constrained to their narrow ranges (Table S1). In the one-batch method, the posterior distributions of parameters (Figure S5, blue curves) had either similar shapes or mean values to the MLE distributions from the random-sampling method (Figure S5 and Table S1).

Some of the estimated parameter values by the neural networking method were linearly correlated with the mean values of parameter's posterior distributions obtained from the site-by-site data assimilation (Table S1 and Figure S6). Well-constrained parameters from the site-by-site and one-batch methods (*efloding, fs1s2, fs1s3*, and *fs2s3*) were usually well-predicted by the trained neural network. Those parameters having flat posterior distributions (*tau4l2l3, tau4l1, fs2s1*, and *fs3s1*) after data assimilation usually cannot be predicted well by the neural networking method.

## Discussion

### Toward More Realistic Representations of SOC Distribution

To the best of our knowledge, this study is the first to systematically explore how to best represent SOC distribution in Earth system models, either by conventional data assimilation methods alone or in combination with a deep learning technique. We have demonstrated that massive observational datasets can be assimilated into ESMs. It has long been recognized that large gaps exist between the representation of SOC distribution by ESMs and observations. Although Bayesian data assimilation has been applied to integration of observations with process-oriented models to improve simulation performance at individual sites (Xu et al., 2006; Li et al., 2016), the complexity of ESMs and the computational cost in the MCMC process hindered the progress of using big data to inform models at a continental or global scale. In this study, we took advantage of the matrix representation of CLM5 to obtain the analytical solution of SOC content along the soil depth under a steady state assumption (Xia et al., 2012; Luo et al., 2017; Huang et al., 2018). The analytical solution saves the computational time of the spin-up process and therefore makes the data assimilation feasible for ESMs. Due to the matrix approach to spin-up, the large amount of high-quality obseravtional data on SOC available across the conterminous United States can be assimilated into CLM5 to improve the representation of modeled SOC distributions.

Both data assimilation and deep learning are powerful tools in understanding complex processes of the Earth system from rapidly increasing data. In this study, data assimilation methods (i.e., the one-batch method) successfully corrected considerable overestimation of the total carbon storage, especially in the deep soil layers, by CLM5 with its original parameterization (Figures 3, 4). However, geographical biases still existed across the conterminous United States when we used data assimilation methods alone (Figure 2). The neural networking method, which optimally reproduces spatial patterns of parameters as in those obtained from the site-by-site data assimilation, corrected the geographical biases from the one-batch or random sampling methods and generated the least geographical bias in SOC representation. As soil carbon is among the most significant and vulnerable components of the terrestrial carbon cycle, realistic representation of spatial and vertical SOC distributions by ESMs is essential to evaluate how the Earth system will respond to the unprecedented climate change and human disturbances. Thus, CLM5 with spatially heterogenous parameter values estimated from the neural networking method generates the best representation of SOC and should be used to assess SOC feedbacks to global warming.

### Varied Parameter Values With Environmental Conditions

We have found that it is essential to have spatially heterogeneous parameter values in generating realistic distributions of SOC over the continental scale. Continentally homogeneous parameter values are apparently insufficient in describing the heterogeneity of SOC distribution among different ecosystems (Figure 5). In this study, both the one-batch and random-sampling methods harmonized individual site information to generate the continental posterior probability density functions of parameters for simulating the SOC distribution. These methods captured the general features of the SOC distribution across the conterminous United States and yielded reasonable total SOC stocks (Figure 4), but failed to represent spatial patterns of the SOC distribution (Figure 5). In contrast, the neural networking method generated a SOC distribution with the minimal spatial biases by producing spatially heterogeneous parameter values. Taking advantage of parameter values estimated at individual sites, the neural networking method associated the mean values of parameter's posterior distributions obtained from the site-by-site data assimilation with 60 auxiliary environmental covariates. The spatially heterogeneous parameter values optimized from the trained neural network contributed to the more accurate estimation of SOC distribution by CLM5 than the other methods.

Although a text-book doctrine of simulation modeling is that parameter values must be constant (Forrester, 1961), constant parameter values may not be sufficient to describe the enormous heterogeneity of carbon cycle dynamics (Luo et al., 2003). For instance, in a study of assimilating soil data into a forest ecosystem carbon model, constrained parameters representing allocation and turnover processes in the soil carbon cycle presented significant variation in different climate zones across China (Ge et al., 2018). In another study, using observational SOC data to retrieve the global pattern of temperature sensitivity of soil respiration (*Q*_10_), after data assimilation, the results showed spatially heterogenous *Q*_10_ values across the globe (Zhou et al., 2009). Constant parameter values in ESMs may lead to strong biases in regional or global simulation of the carbon cycle. Future research may further explore whether or not and how parameter values may vary over space for SOC dynamics.

### Terrestrial Carbon Cycle Studies in the Big Data Era

Data assimilation techniques bridge the gap between the simulation of process-oriented models and observations. ESMs informed by observations through data assimilation present more realistic simulations on soil carbon dynamics than the conventional *ad hoc* parameterization (Hararuk et al., 2014). However, limited data sets may not constrain all the parameters as shown in this study (Table S1 and Figure S5). Uncertainties from the unconstrained parameters will be propagated to the projection of SOC dynamics in future scenarios (Luo et al., 2009, 2016). On one hand, we need more types of high-quality observations to constrain more parameters in ESMs. On the other hand, we may never collect enough data sets to constrain all the parameters in complex models such as CLM5. It becomes a philosophical question of how complex a model is supposed to be so that it can capture enough processes in a system yet be constrained by observations. Data assimilation offers information on which parameters can and cannot be constrained by a given set of observations. Such information can inform the development of ESMs.

Deep learning, another technique we used in this study, is a powerful tool that can be used to extract patterns from big data. Several studies have applied deep learning techniques to emulate ESM output, drive submodels in Earth system modeling, and evaluate model-observation mismatches (Reichstein et al., 2019). One of the most relevant applications of deep learning for this study is to generate static spatially-explicit soil maps for model input or evaluation. However, knowledge of how to combine deep learning with process understanding of biogeochemical cycles in ESMs is still in its infancy. As a pioneer study in this field, our study combined deep learning with data assimilation to retrieve the most realistic SOC distribution by CLM5. The successful representation of SOC distribution across the conterminous United States by the neural networking method points toward a novel direction in hybridizing deep learning with process-oriented modeling for better projections of terrestrial carbon dynamics at regional and global scales. In this study, the high agreement between observed and modeled SOC content (*R*^2^ = 0.623 across the conterminous United States) achieved by the hybrid data assimilation-deep learning was comparable with that for harmonization mapping in SoilGrids250m by machine learning (*R*^2^ = 0.635 across the globe) (Hengl et al., 2017), and greater than the agreement between separate gridded empirical data products (Wu et al., 2019). However, the SOC mapping provides only the static spatial patterns of SOC, which differ greatly among different data products (Tifafi et al., 2018; Dai et al., 2019). This version of CLM5 that is constrained by big data of SOC vertical profiles and optimized by deep learning enables us to not only map SOC distributions but also examine the spatiotemporal dynamics of SOC in response to climate change.

In summary, our study integrated deep learning, data assimilation, and a large amount of high-quality observational data to improve the representation of SOC distribution in a land biogeochemical model (CLM5) across the conterminous United States Our results indicated that the *ad hoc* parameterization in CLM5 cannot adequately represent SOC content spatially or vertically. CLM5 with default parameter values dramatically overestimated the total SOC storage of the conterminous United States and presents severe geographical biases in the representation of SOC distribution. Constrained parameter values after data assimilation improved SOC storage estimates in CLM5 and yielded better spatial and vertical distributions of SOC than the original model. Deep learning hybridized with data assimilation identified the spatially heterogenous parameters across the conterminous United States The spatial variation in the parameterization of CLM5 further improves the quantification of SOC in different ecosystems with the highest agreement and least bias in comparison with observations. The realistic representation of SOC distribution via data assimilation and deep learning techniques pave a way for a more accurate evaluation of terrestrial carbon dynamics and its feedbacks to climate change in ESMs.

## Data Availability Statement

All the codes and data presented in this research are available upon request to the corresponding author.

## Author Contributions

YLu and FT designed this study. FT and ZZ collected and cleansed the data and performed the site-by-site method and random sampling method. FT performed the one-batch method and neural networking method. XH and YLi contributed to conducting the neural networking and high-performance computing used in this research. RD and ZR contributed to collecting NPP and soil data. FT, YLu, YH, and QL drafted the first versions of the manuscript. All the co-authors reviewed and contributed to the revisions of the manuscript.

## Conflict of Interest

The authors declare that the research was conducted in the absence of any commercial or financial relationships that could be construed as a potential conflict of interest. The reviewer DW declared a shared affiliation, with no collaboration, with one of the authors, XL, to the handling editor at the time of review.
