# Supplementary material for: Deep Learning Optimizes Data-Driven Representation of Soil Organic Carbon in Earth System Model Over the Conterminous United States
Source: Front Big Data. 2020 Jun 3;3:17. doi: 10.3389/fdata.2020.00017 (PMC7931903; doi:10.3389/fdata.2020.00017)
Supplement: Supplementary file 1 [file Data_Sheet_1.pdf]

Supplementary Material

Computing Intensity of Data Assimilation

Large computational cost arising from the complexity of CLM5 has largely been solved by the matrix representation of the soil carbon cycle (Luo et al., 2017). By assuming a steady state and given the average of multi-year carbon input and climate variables, an analytical solution of SOC content can be calculated without lengthy spin-up process (Niu et al., 2014; Weng & Luo, 2011; Xu, White, Hui, & Luo, 2006; T. Zhou & Luo, 2008; X. Zhou, Zhou, & Luo, 2012). The time of processing one soil profile in a personal computer (processor: 2.6 GHz Intel Core i5, Memory: 8 GB 1600 MHz DDR3) was 0.5 to 1 hour. The parallel computing toolbox provided by MATLAB R2017b was applied to accelerate the computation. By operating codes on a server which contains 20 nodes (24 cores for each node) provided by National Supercomputing Center in Wuxi (NSCCWX), China, the total time of processing all soil profiles in site-by-site data assimilation was around 30 hours. The one-batch method was operated based on a FORTRAN script. Message Passing Interface (MPI) was applied to improve computing efficiency. By using one node containing 24 cores of the server provided by NSCCWX, the one-batch data assimilation can be finished within 48 hours.

All the codes relating to the site-by-site and random-sampling methods were written by MATLAB R2017b, the neural network was built by Keras in Python. The code of one-batch data assimilation was written by FORTRAN. Data analyses and figures were constructed by R language. All the codes can be accessed on request.

Supplementary Figures and Tables

Table S1. Parameters used in describing the dynamics of carbon in litter and soil in CLM5 and statistics in data assimilation methods and the neural networking method. The matrix source indicates the part that the parameter is used in Eq. 1. The initial values and ranges are used in MCMC as a priori. NA indicates the value of parameters is not constant in the original model. In site-by-site data assimilation, Maximum Likelihood Estimates (MLE) of parameter's posterior distributions were calculated at individual sites. The mean values of MLE for each parameter were then presented. The Gelman-Rubin statistic of the three parallel MCMC chains and the variance of the parameters' posterior distribution in site-by-site data assimilation are also listed. SD indicates the standard deviation. In neural networking method, the Pearson correlation and corresponding p-value between predicted values and the mean values of parameters' posterior distribution in site-by-site data assimilation are reported. All parameters values were normalized to the interval of [0, 1], based on the prior ranges. All the statistics related to variance were calculated based on the normalized results of parameters.

							Site-by-Site Data Assimilation					One-Batch Data Assimilation			Neural Networking	
**No.**	**Name**	**Matrix Source**	**Description**	**Initial Value**	**Unit**	**Prior Range**	**Mean MLE Value**	**Mean G-R Statistic**	**G-R SD**	**Mean Variance of Posterior Distribution**	**Variance of SD**	**Posterior Mean Value**	**Variance of Posterior Distribution**	**G-R Statistic**	**Correlation in Prediction**	**p-value**
1	***efolding***	ξ	e-folding parameter for depth scalar	0.5		[0 1]	0.77	1.038	0.080	0.022	0.010	0.81	0.017	1.00	0.44	0
2	***tau4s3***	**K**	Turnover time of passive SOC	222	yr	[200 2000]	486	1.108	0.149	0.026	0.020	486	0.022	1.00	0.42	0
3	***tau4s2***	**K**	Turnover time of slow SOC	5	yr	[1 50]	11.91	1.097	0.181	0.025	0.022	15.65	0.041	1.00	0.61	0
4	***q10***	ξ	Temperature sensitivity	1.5		[1.2 3]	1.51	1.047	0.097	0.039	0.021	1.40	0.0093	1.00	0.72	0
5	***fs1s3***	**A**	Transfer fraction, fast SOC to passive SOC	NA		[0 0.03]	0.0045	1.072	0.106	0.027	0.024	0.0070	0.036	1.00	0.61	0
6	***fs2s3***	**A**	Transfer fraction, slow SOC to passive SOC	0.037		[0 0.1]	0.015	1.082	0.118	0.029	0.025	0.025	0.044	1.00	0.54	0
7	***ins***	**I**	Input scalar	1		[NPP_{min}/NPP_{mean} NPP_{max}/NPP_{mean}]	0.92	1.039	0.082	0.058	0.016	1.10	0.029	1.00	0.52	0
8	***diffus***	**V**	Bioturbation rate	0.0001	m^2yr^{-1}	[3×10^{-5} 5×10^{-4}]	0.00023	1.071	0.171	0.037	0.021	0.00028	0.056	1.00	0.51	0
9	***fs1s2***	**A**	Transfer fraction, fast SOC to slow SOC	NA		[0 0.4]	0.11	1.063	0.109	0.047	0.018	0.12	0.051	1.00	0.57	0
10	***beta***	**I**	Beta parameter controlling vertical distribution of litter input	NA		[0.5 1]	0.72	1.051	0.734	0.041	0.019	0.71	0.056	1.00	0.50	0
11	***p4ll***	**I**	Fraction from NPP to lignin litter	NA		[0 0.25]	0.092	1.045	0.089	0.061	0.011	0.095	0.062	1.00	0.47	0
12	***p4ml***	**I**	Fraction from NPP to metabolic litter	NA		[0.05 0.7]	0.35	1.035	0.081	0.059	0.007	0.34	0.064	1.01	0.33	0
13	***fl3s2***	**A**	Transfer fraction, lignin litter to slow SOC	0.5		[0.2 0.8]	0.41	1.037	0.080	0.061	0.010	0.42	0.064	1.00	0.51	0

14	***fl2s1***	**A**	Transfer fraction, cellulose litter to fast SOC	0.5		[0.2 0.8]	0.43	1.035	0.075	0.065	0.009	0.43	0.065	1.00	0.42	0
15	***maxpsi***	**ξ**	Maximum soil water potential	-0.002	MPa	[-0.015 0]	-0.0088	1.026	0.062	0.064	0.007	-0.0084	0.068	1.00	0.23	0
16	***tau4s1***	**K**	Turnover time of fast SOC	0.14	yr	[0 1]	0.58	1.033	0.080	0.064	0.012	0.51	0.071	1.00	0.40	0
17	***p4cl***	**I**	Fraction from NPP to cellulose litter	NA		[0.01 0.5]	0.24	1.029	0.062	0.065	0.007	0.24	0.068	1.00	0.27	0
18	***fl1s1***	**A**	Transfer fraction, metabolic litter to fast SOC	0.45		[0.2 0.8]	0.45	1.033	0.086	0.067	0.008	0.45	0.068	1.00	0.39	0
19	***fcwdl2***	**A**	Transfer fraction, CWD to cellulose litter	0.76		[0.5 1]	0.81	1.035	0.077	0.067	0.008	0.77	0.072	1.00	0.40	0
20	***tau4l1***	**K**	Turnover time of metabolic litter	0.054		[0 0.2]	0.11	1.027	0.064	0.070	0.006	0.099	0.072	1.00	0.051	0.0007
21	***tau4l2l3***	**K**	Turnover time of cellulose and lignin litter	0.20	yr	[0.2 1]	0.62	1.027	0.064	0.070	0.007	0.60	0.073	1.00	0.23	0
22	***cryo***	**V**	Cryoturbation rate	0.0001	m^2yr^{-1}	[3×10^{-5} 16×10^{-4}]	0.00088	1.027	0.064	0.071	0.006	0.00081	0.073	1.00	0.035	0.021
23	***fs2s1***	**A**	Transfer fraction, slow SOC to fast SOC	0.51		[0.1 0.6]	0.34	1.028	0.068	0.070	0.006	0.34	0.073	1.00	0.13	0
24	***tau4cwd***	**K**	Turnover time of CWD	3.33	yr	[1 10]	5.87	1.027	0.062	0.071	0.006	5.64	0.073	1.00	0.034	0.021
25	***fs3s1***	**A**	Transfer fraction, passive SOC to fast SOC	0.45		[0 1]	0.54	1.027	0.061	0.071	0.006	0.48	0.073	1.00	0.044	0.003

Table S2. Auxiliary environmental covariates used in the neural networking method to predict parameter values of CLM5 for each observation site and each grid of the US continent.

No.	Variable Name	Data Source	Resolution (degree)	Description
1	Climate Types	(Kottek, Grieser, Beck, Rudolf, & Rubel, 2006)	0.5	
2	Soil Types	FAO-UNESCO	0.083	
3	NPP	(DAAC, 2018; Zhao, Heinsch, Nemani, & Running, 2005; Zhao & Running, 2010; Zhao, Running, & Nemani, 2006)	0.5	Three subsets were included, which are mean annual NPP, maximum annual NPP, and minimum annual NPP. Mean value of the record from 2000 to 2014
4	Vegetation Cover	(Channan, Collins, & Emanuel, 2014; Friedl et al., 2010)	0.5	
5	Annual Mean Temperature	(Fick & Hijmans, 2017)	0.5	Mean value of the record from 1970 to 2000
6	Mean Diurnal Range Temperature	(Fick & Hijmans, 2017)	0.5	Mean value of the record from 1970 to 2000
7	Isothermality	(Fick & Hijmans, 2017)	0.5	Mean value of the record from 1970 to 2000
8	Temperature Seasonality	(Fick & Hijmans, 2017)	0.5	Mean value of the record from 1970 to 2000
9	Max Temperature of Warmest Month	(Fick & Hijmans, 2017)	0.5	Mean value of the record from 1970 to 2000
10	Min Temperature of Coldest Month	(Fick & Hijmans, 2017)	0.5	Mean value of the record from 1970 to 2000
11	Temperature Annual Range	(Fick & Hijmans, 2017)	0.5	Mean value of the record from 1970 to 2000
12	Mean Temperature of Wettest Quarter	(Fick & Hijmans, 2017)	0.5	Mean value of the record from 1970 to 2000

13	Mean Temperature of Driest Quarter	(Fick & Hijmans, 2017)	0.5	Mean value of the record from 1970 to 2000
14	Mean Temperature of Warmest Quarter	(Fick & Hijmans, 2017)	0.5	Mean value of the record from 1970 to 2000
15	Mean Temperature of Coldest Quarter	(Fick & Hijmans, 2017)	0.5	Mean value of the record from 1970 to 2000
16	Annual Precipitation	(Fick & Hijmans, 2017)	0.5	Mean value of the record from 1970 to 2000
17	Precipitation of Wettest Month	(Fick & Hijmans, 2017)	0.5	Mean value of the record from 1970 to 2000
18	Precipitation of Driest Month	(Fick & Hijmans, 2017)	0.5	Mean value of the record from 1970 to 2000
19	Precipitation Seasonality	(Fick & Hijmans, 2017)	0.5	Mean value of the record from 1970 to 2000
20	Precipitation of Wettest Quarter	(Fick & Hijmans, 2017)	0.5	Mean value of the record from 1970 to 2000
21	Precipitation of Driest Quarter	(Fick & Hijmans, 2017)	0.5	Mean value of the record from 1970 to 2000
22	Precipitation of Warmest Quarter	(Fick & Hijmans, 2017)	0.5	Mean value of the record from 1970 to 2000
23	Precipitation of Coldest Quarter	(Fick & Hijmans, 2017)	0.5	Mean value of the record from 1970 to 2000
24	Absolute Depth to Bedrock	(Hengl et al., 2017)	0.01	
25	USDA 2014 Suborder Classes	(Hengl et al., 2017)	0.01	
26	WRB 2006 Subgroup Classes	(Hengl et al., 2017)	0.01	
27	Texture Classes	(Hengl et al., 2017)	0.01	Three depths were included, which are 0cm, 30cm and 100cm, respectively
28	Sand Content	(Hengl et al., 2017)	0.01	Three depths were included, which are 0cm, 30cm and 100cm, respectively
29	Soil pH in H_2O	(Hengl et al., 2017)	0.01	Three depths were included, which are

				0cm, 30cm and 100cm, respectively
30	Bulk Density	(Hengl et al., 2017)	0.01	Three depths were included, which are 0cm, 30cm and 100cm, respectively
31	Coarse Fragments Volumetric	(Hengl et al., 2017)	0.01	Three depths were included, which are 0cm, 30cm and 100cm, respectively
32	Clay Content	(Hengl et al., 2017)	0.01	Three depths were included, which are 0cm, 30cm and 100cm, respectively
33	Silt Content	(Hengl et al., 2017)	0.01	Three depths were included, which are 0cm, 30cm and 100cm, respectively
34	Soil pH	(Hengl et al., 2017)	0.01	Three depths were included, which are 0cm, 30cm and 100cm, respectively
35	Soil Water Capacity	(Hengl et al., 2017)	0.01	Three depths were included, which are 0cm, 30cm and 100cm, respectively
36	Cation Exchange Capacity	(Hengl et al., 2017)	0.01	Three depths were included, which are 0cm, 30cm and 100cm, respectively
37	Depth to Bedrock (R horizon)	(Hengl et al., 2017)	0.01	
38	Grade of a Sub-Soil Being Acid	(Hengl et al., 2017)	0.01	
39	Palmer Drought Severity Index (PDSI)	(Dai, Trenberth, & Qian, 2004)	0.5	The original resolution was 2.5 degree and we resampled this dataset to 0.5 degree. Mean value of the record from 1870 to 2002

Table S3. SOC storage of the U.S. continent along the soil depth estimated by different methods and data sources. Values in the brackets are 5% and 95% quantile of the simulation results in the random sampling method and the one batch method.

Methods	Estimated SOC Storage (Pg C)		
	0 - 30cm	30 - 100cm	100 - 200cm
**WISE30sec**	**97.7**	**80.8**	**63.8**
**SoilGrids250m**	**102.4**	**86.4**	**80.7**
**Default**	**157.6**	**367.8**	**525.5**
**Random Sampling**	**84.1 (10.7, 263.4)**	**62.6 (4.3, 197.2)**	**57.6 (1.8, 162.8)**
**One Batch**	**77.4 (49.0, 112.1)**	**52.4 (34.0, 74.0)**	**36.2 (23.7, 51.0)**
**Neural Networking**	**101.2**	**77.3**	**68.0**

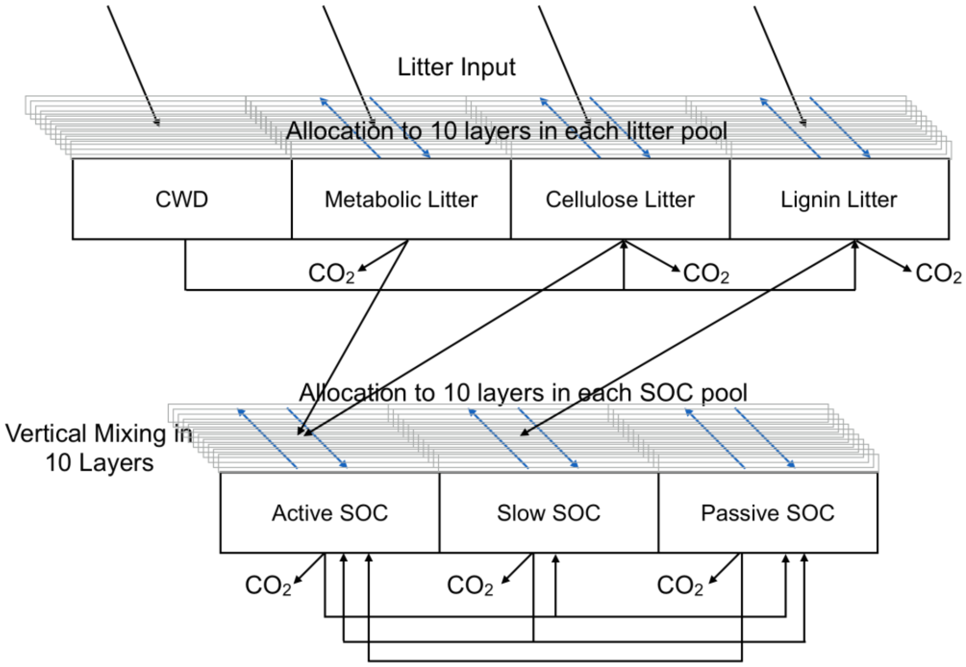

Figure S1. Carbon dynamics in litter and soil pools in CLM5. Four aboveground carbon pools are classified in CLM5, which are coarse woody debris (CWD), metabolic litter, cellulose litter, and lignin litter, respectively. In addition to the organic carbon keep in litter pools, carbon fluxes are either released to atmospheric by respiration as CO_2 or transferred to soil carbon pools (as shown by arrows). Three soil carbon pools are tracked in CLM5, which are identified by active, slow and passive SOC, respectively. Carbon fluxes in soil carbon pools can be stored in the pool for a period of time or be released to the atmosphere by the form of CO_2 through respiration or be transferred between two soil carbon pools. Meanwhile, vertical mixing happens in different soil layers in each litter and soil carbon pools by cryoturbation or bioturbation. The schematic figure is adapted from the work of Huang et al. (Huang et al., 2018).

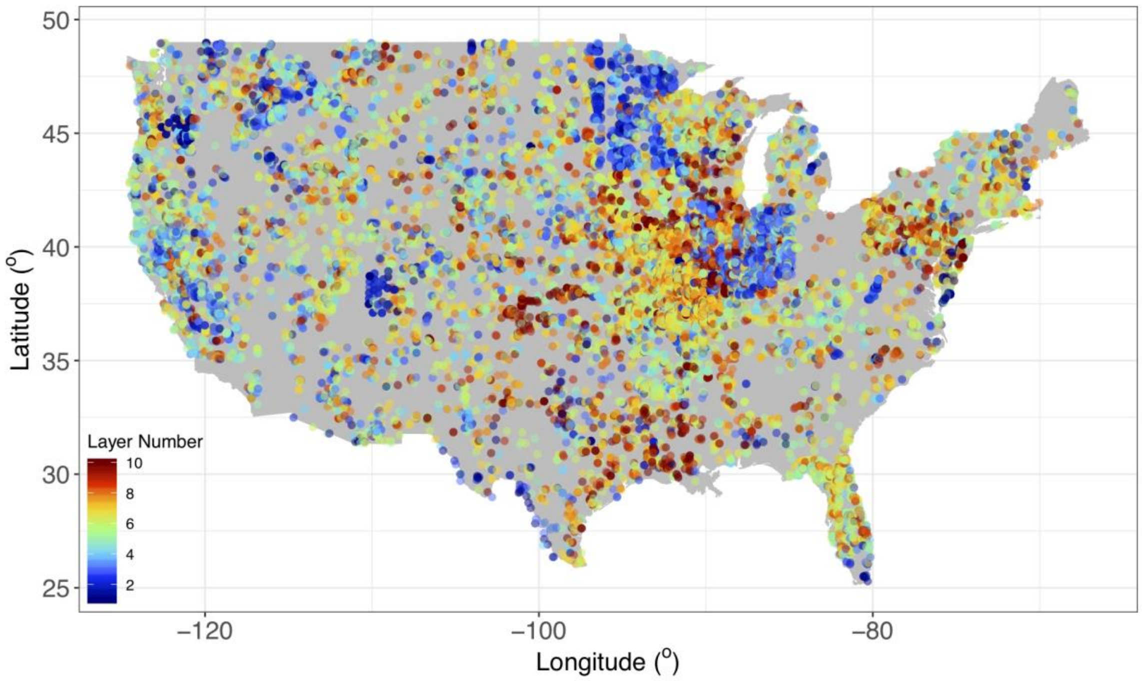

Figure S2. The geographical location and number of soil layers for each soil profile used in this study. The color represents the number of soil layers at the corresponding site.

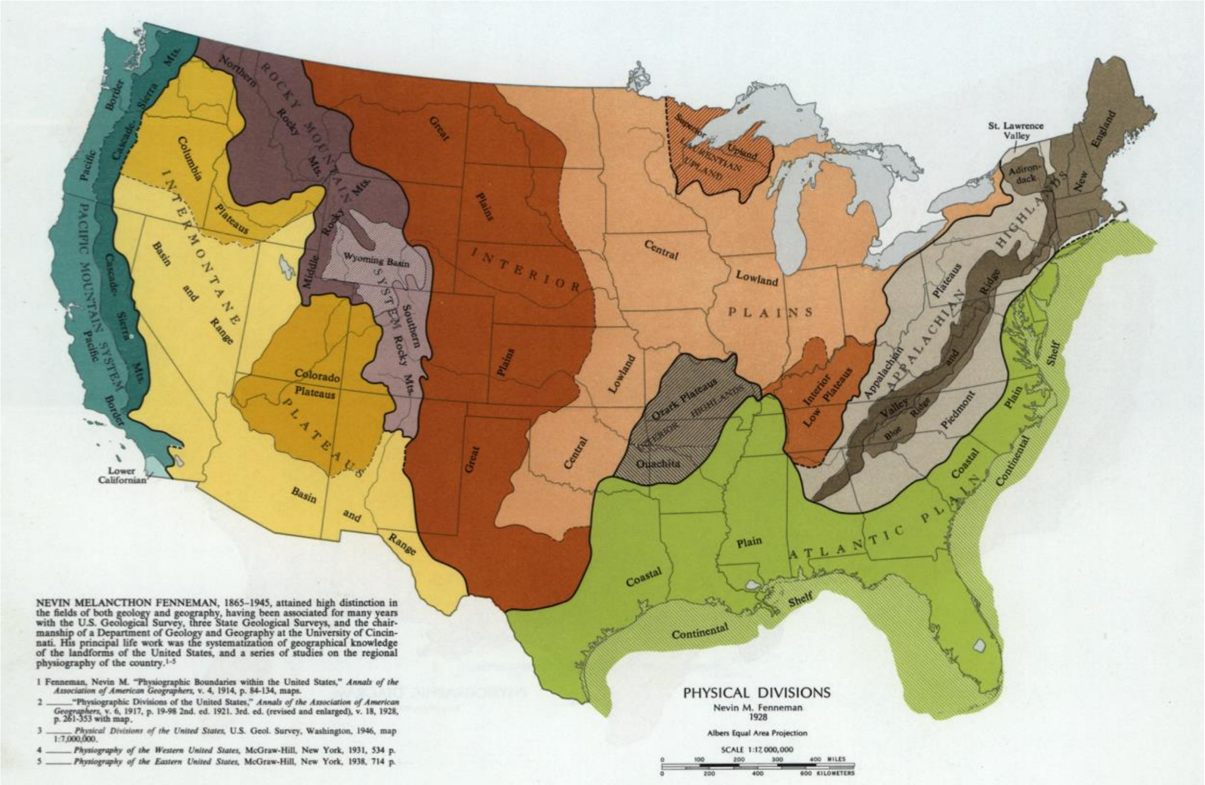

Figure S3. Physiographical regions of the conterminous United States. The regions colored by yellow are the Intermontane Plateaus. The regions colored by dark brown are the Great Plains. Image is from the Library of Congress, "*The national atlas of the United States of America*". Source link: https://www.loc.gov/resource/g3701gm.gct00013/?sp=46&r=-0.182,0.635,1.448,0.702,0.

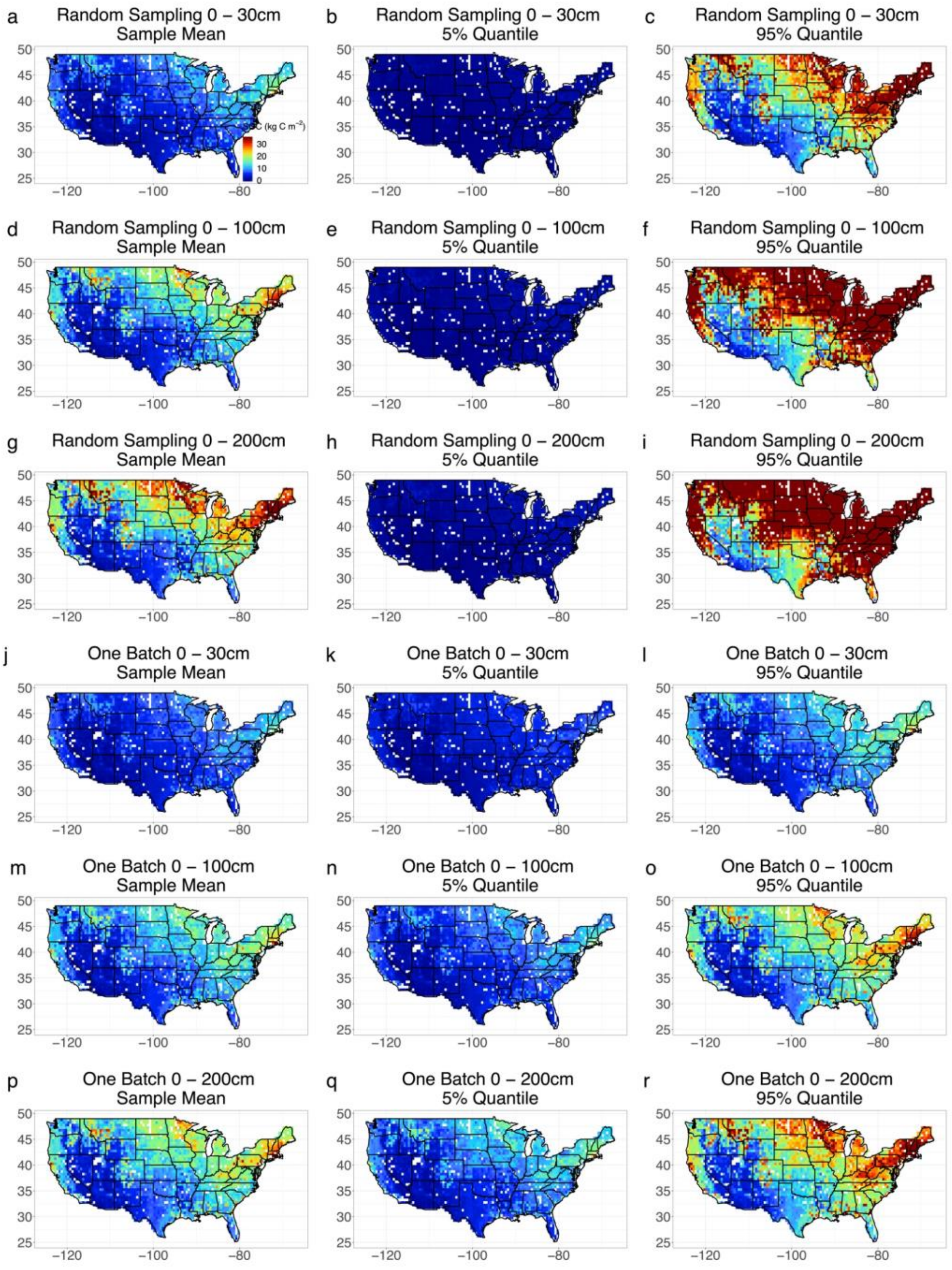

Figure S4. Simulation uncertainties in the random-sampling and one-batch methods. Figures in the frist column are the mean values from the 1,000-time simulations. Firgures in the second and third column are the 5% and 95% quantiles of the simulation results.

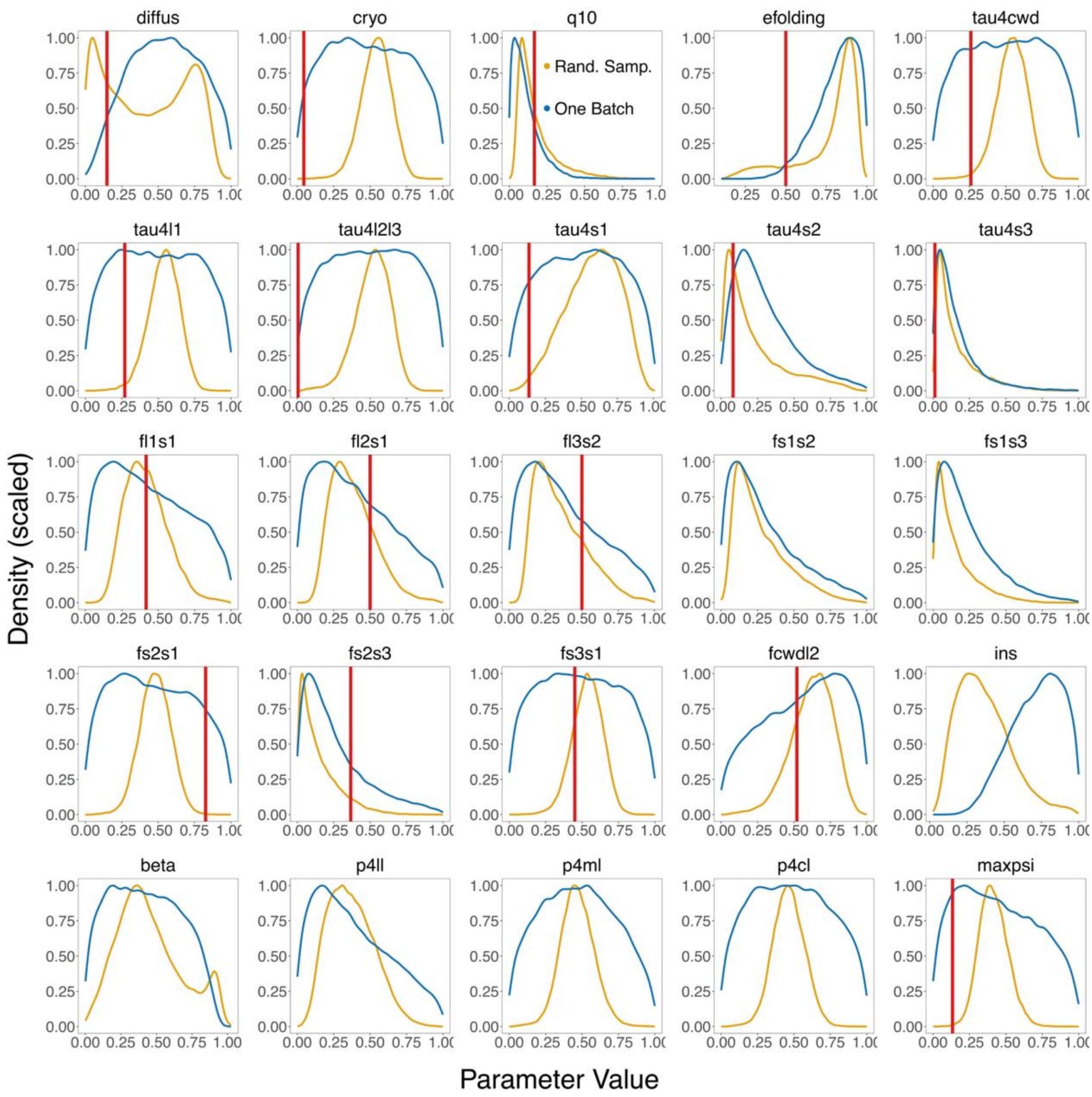

Figure S5. Posterior distributions for each parameter in CLM5 derived from the random-sampling method (yellow line) and the one-batch method (blue line). The red vertical line represents the default value in the original CLM5 model. For parameters without showing the default values, the parameter values in original CLM5 are either different among different soil textures (e.g., *fs1s2* and *fs1s3*) or vegetation types (e.g., *beta*) or varied among different simulation years (e.g., *ins*, *p4ll*, *p4ml*, *p4cl*).

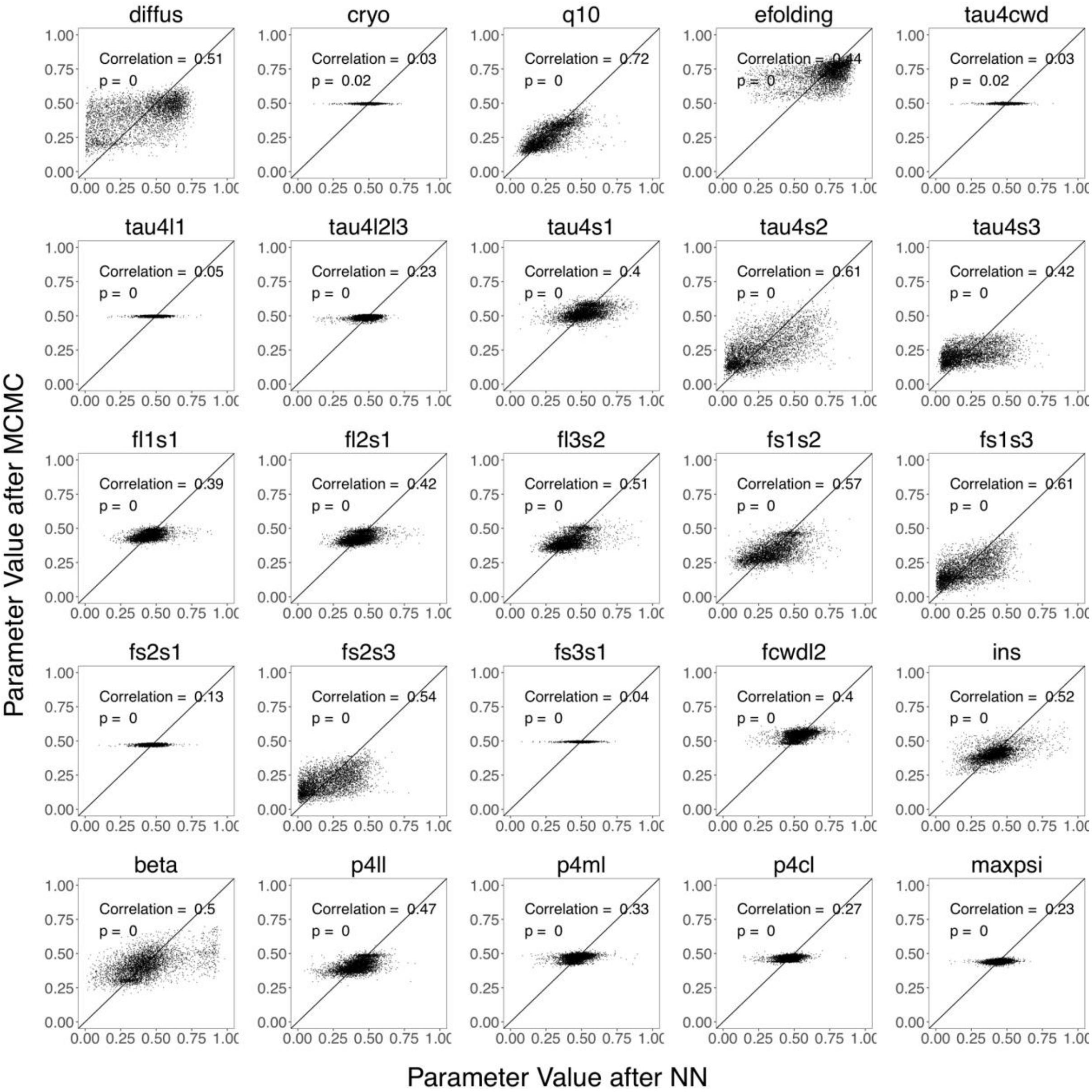

Figure S6. Predicted parameters by the trained neural network in the neural networking method. Note, only the results in the testing set were presented.

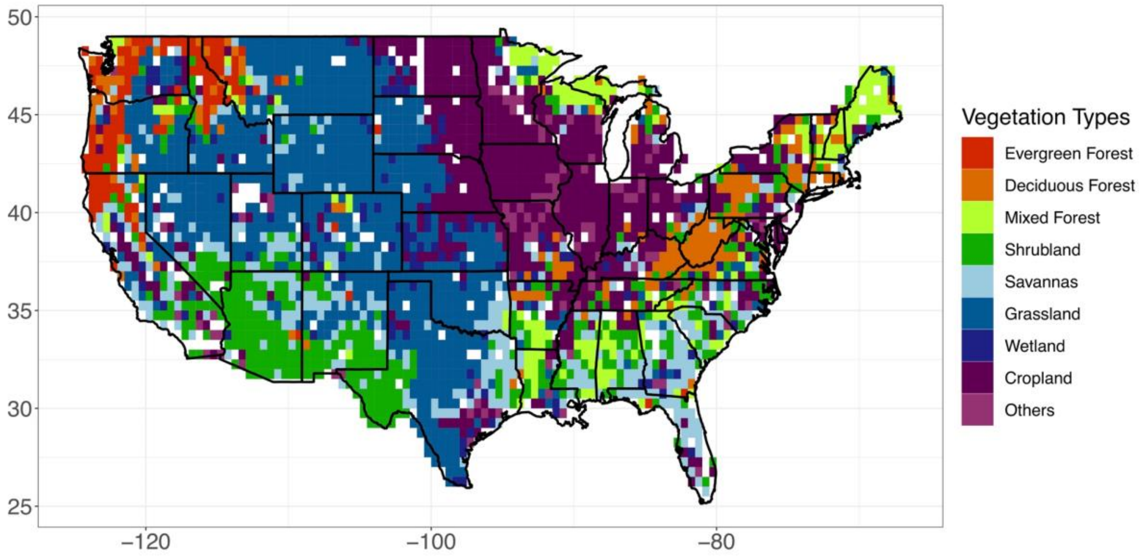

Figure S7. The distribution of vegetation types of the U.S. continent. The data source is as listed in Table S2

References:

Channan, S., Collins, K., & Emanuel, W. (2014). Global mosaics of the standard MODIS land cover type data. *University of Maryland and the Pacific Northwest National Laboratory, College Park, Maryland, USA, 30*.

DAAC, N. L. (2018). *Terra MODIS Net Primary Production Yearly L4 Global 1km*.

Dai, A., Trenberth, K. E., & Qian, T. (2004). A global dataset of Palmer Drought Severity Index for 1870–2002: Relationship with soil moisture and effects of surface warming. *Journal of Hydrometeorology, 5*(6), 1117-1130.

Fick, S. E., & Hijmans, R. J. (2017). WorldClim 2: new 1-km spatial resolution climate surfaces for global land areas. *International Journal of Climatology, 37*(12), 4302-4315.

Friedl, M. A., Sulla-Menashe, D., Tan, B., Schneider, A., Ramankutty, N., Sibley, A., et al. (2010). MODIS Collection 5 global land cover: Algorithm refinements and characterization of new datasets. *Remote sensing of Environment, 114*(1), 168-182.

Hengl, T., de Jesus, J. M., Heuvelink, G. B., Gonzalez, M. R., Kilibarda, M., Blagotić, A., et al. (2017). SoilGrids250m: Global gridded soil information based on machine learning. *PLoS One, 12*(2), e0169748.

Huang, Y., Lu, X., Shi, Z., Lawrence, D., Koven, C. D., Xia, J., et al. (2018). Matrix approach to land carbon cycle modeling: A case study with the Community Land Model. *Global change biology, 24*(3), 1394-1404.

Kottek, M., Grieser, J., Beck, C., Rudolf, B., & Rubel, F. (2006). World map of the Köppen-Geiger climate classification updated. *Meteorologische Zeitschrift, 15*(3), 259-263.

Luo, Y., Shi, Z., Lu, X., Xia, J., Liang, J., Jiang, J., et al. (2017). Transient dynamics of terrestrial carbon storage: mathematical foundation and its applications. *Biogeosciences, 14*(1), 145.

Niu, S., Luo, Y., Dietze, M. C., Keenan, T. F., Shi, Z., & Li, J. (2014). The role of data assimilation in predictive ecology. *Ecosphere, 5*(5), 1-16.

Weng, E., & Luo, Y. (2011). Relative information contributions of model vs. data to short-and long-term forecasts of forest carbon dynamics. *Ecological Applications, 21*(5), 1490-1505.

Xu, T., White, L., Hui, D., & Luo, Y. (2006). Probabilistic inversion of a terrestrial ecosystem model: Analysis of uncertainty in parameter estimation and model prediction. *Global biogeochemical cycles, 20*(2).

Zhao, M., Heinsch, F. A., Nemani, R. R., & Running, S. W. (2005). Improvements of the MODIS terrestrial gross and net primary production global data set. *Remote sensing of Environment, 95*(2), 164-176.

Zhao, M., & Running, S. W. (2010). Drought-induced reduction in global terrestrial net primary production from 2000 through 2009. *science, 329*(5994), 940-943.

Zhao, M., Running, S. W., & Nemani, R. R. (2006). Sensitivity of Moderate Resolution Imaging Spectroradiometer (MODIS) terrestrial primary production to the accuracy of meteorological reanalyses. *Journal of Geophysical Research: Biogeosciences, 111*(G1).

Zhou, T., & Luo, Y. (2008). Spatial patterns of ecosystem carbon residence time and NPP-driven carbon uptake in the conterminous United States. *Global biogeochemical cycles, 22*(3).

Zhou, X., Zhou, T., & Luo, Y. (2012). Uncertainties in carbon residence time and NPP-driven carbon uptake in terrestrial ecosystems of the conterminous USA: a Bayesian approach. *Tellus B: Chemical and Physical Meteorology, 64*(1), 17223.
